# Chromosome-scale genome assembly and genomic prediction of essential oil compounds in *Atractylodes lancea* for genomics-assisted breeding

**DOI:** 10.1093/dnares/dsag014

**Published:** 2026-09-10

**Authors:** Takahiro Tsusaka, Kenta Shirasawa, Yunjia Zhang, Ting-Fung Chan, Hiroki Yamaji, Sachiko Isobe

**Affiliations:** Botanical Raw Materials Cultivation Technology Development Department, Tsumura & Co., Ami, Ibaraki 300-1192, Japan; Department of Frontier Research and Development, Kazusa DNA Research Institute, Chiba 292-0818, Japan; School of Life Sciences, The Chinese University of Hong Kong, Shatin, Hong Kong; The State Key Laboratory of Agrobiotechnology, The Chinese University of Hong Kong, Shatin, Hong Kong; School of Life Sciences, The Chinese University of Hong Kong, Shatin, Hong Kong; The State Key Laboratory of Agrobiotechnology, The Chinese University of Hong Kong, Shatin, Hong Kong; Botanical Raw Materials Cultivation Technology Development Department, Tsumura & Co., Ami, Ibaraki 300-1192, Japan; Department of Frontier Research and Development, Kazusa DNA Research Institute, Chiba 292-0818, Japan; Graduate School of Agricultural and Life Sciences, University of Tokyo, Tokyo 113-8657, Japan

**Keywords:** *Atractylodes lancea*, chromosome-scale genome assembly, essential oil compounds, genome-wide association study, genomic prediction

## Abstract

*Atractylodes lancea* rhizomes are used as crude drugs. Essential oil compounds, including atractylodin, hinesol, β-eudesmol, and atractylon, are key determinants of crude drug quality. Conventional breeding of *A. lancea* is difficult because of its perennial growth. In this study, a chromosome-scale reference genome of *A. lancea* (4.79 Gb) was generated, and genome-wide association studies (GWAS) and genomic predictions of essential oil compounds were conducted to explore the potential for genome-assisted breeding. Genotyping of 480 lines using double-digest restriction-site–associated DNA-sequencing yielded 29,136 high-quality SNPs. All the compounds showed high genomic heritability (*h*^2^ = 0.758–0.915), indicating strong genetic control. Despite the high genomic heritability, GWAS detected only one weak association with atractylon and no significant loci for the three compounds. However, genomic prediction achieved moderate to high accuracy across multiple models, particularly the ridge regression, genomic best linear unbiased prediction, and Bayesian approaches. The prediction accuracy, measured as the Pearson correlation coefficient between the observed and predicted values, exceeded 0.6 for all four essential oil compounds. These results demonstrate the efficacy of genomic selection for improving essential oil compound levels in *A. lancea* and provide a foundation for genome-assisted breeding of medicinal plants with long breeding cycles.

## 1. Introduction

*Atractylodes lancea* De Candolle is a perennial medicinal plant belonging to the family Asteraceae. It is native to East Asia, primarily distributed in central China.^1^ The dried rhizomes of *A. lancea* are used as crude drugs in traditional Chinese and Japanese medicine, mainly for the treatment of digestive disorders and imbalances in body fluid homeostasis.^2,3^ Essential oil compounds, including sesquiterpenoids and polyacetylenes, are recognized as the major bioactive constituents of *A. lancea*. In particular, β-eudesmol, hinesol, and atractylodin have been reported to exhibit various pharmacological activities.^4^ β-Eudesmol and hinesol have been shown to possess anti-ulcer activity, whereas atractylodin has been reported to ameliorate delayed gastric emptying.^5,6^ Atractylon is detected in *A. lancea* but is a major essential oil constituent of closely related species such as *A. japonica* and *A. macrocephala*, making it a useful chemotaxonomic marker for species discrimination.^7^ Previous research revealed that essential oil compounds in *A. lancea* play a crucial role in determining the quality of crude drugs used as medicinal raw materials.

The levels of essential oil compounds in *A. lancea* vary substantially depending on their natural habitats.^8^ In addition, wild resources of *A. lancea* have declined significantly due to anthropogenic pressures, and large-scale cultivation has expanded in recent years. Consequently, the levels of these compounds in field-cultivated *A. lancea* are often reduced compared with those in wild populations, thereby adversely affecting the quality of the crude drug.^9^ Therefore, controlling the levels of these compounds is crucial for maintaining the quality of *A. lancea* during cultivation.

Thus far, we have investigated the effects of selective breeding on the stabilization of essential oil compound levels using clonal lines of *A. lancea* grown under micro- and macro-environmental conditions.^10^ Our analyses revealed that the broad-sense heritability of essential oil compound levels was high, ranging from 0.77 to 0.86. Furthermore, when clonal lines of *A. lancea* were cultivated at two different locations, there were strong positive correlations in the essential oil compound levels between the sites. These results indicate that the genetic potential underlying essential oil compound accumulation in *A. lancea* is largely stable across different environmental conditions.^10^ Accordingly, selective breeding is an effective strategy for controlling the quality of essential oil compounds in this species. However, the development of elite *A. lancea* cultivars through conventional breeding remains time-consuming, as the evaluation of key traits, including essential oil level and medicinal rhizome yield, typically requires several years owing to the plant's perennial nature and slow growth rate.^11,12^ This long breeding cycle is a major bottleneck in the genetic improvement of *A. lancea*.

Advances in next-generation sequencing technologies have enabled de novo genome assembly and transcriptome analysis in non-model plant species, including medicinal plants.^13,14^ In *A. lancea*, genome sequence and gene annotation data have been generated, and a chromosome-scale genome assembly has been reported.^15,16^ Moreover, genome-wide association analyses have been conducted in *A. lancea* to identify genomic regions associated with metabolite content.^15,16^ Furthermore, transcriptome-based analyses have identified candidate transcription factors, such as bHLH and WRKY proteins, potentially involved in the regulation of sesquiterpenoid biosynthesis in *A. lancea*.^17,18^ However, because the genome sequence data reported in previous studies are not directly available through public sequence databases, their immediate use as reference genomes for downstream analyses is limited. In addition, although genomic regions and genes associated with the contents of bioactive compounds have been explored in *A. lancea*, the development of DNA markers and the assessment of genomic prediction models for breeding have not yet been conducted.

In this study, we generated a chromosome-scale reference genome for our *A. lancea* breeding population and used it to conduct genome-wide association analyses and evaluate genomic prediction models for essential oil compound levels, aiming to develop efficient and effective breeding strategies.

## 2. Materials and Methods

### 2.1. Plant materials and cultivation

In this study, seeds of *A. lancea* were obtained from plant materials used in previous studies^10,12^ These source materials included clonal lines of different origins that had been maintained by vegetative propagation and characterized in previous studies.^12^ The 480 lines used in the present study were generated as seed-derived progeny obtained under natural breeding conditions from this source population and were subsequently propagated using rhizomes derived from single individuals. Each line was assigned a unique identification label (TK17_XXX), which was consistently used across phenotypic and genotypic datasets. A complete list of line identifiers and their inclusion in downstream analyses is provided in Table S1. Rhizomes from each line were divided into approximately 50-g segments and cultivated for one year in an experimental field located in Ami-machi, Inashiki-gun, Ibaraki Prefecture (35°59′ N, 140°12′ E), Japan, in 2018. After cultivation, the rhizomes were harvested and air-dried at 50 °C for 7 days in a drying oven prior to chemical analyses. Phenotypic measurements were performed for each line using a single biological replicate (*n* = 1). This experimental design was adopted because a previous study demonstrated high heritability and high reproducibility of the target traits in *A. lancea*.^10^ Y-T16-69 was selected as the reference genome line because it showed typical morphological characteristics of *A. lancea* and was suitable for stable maintenance through *in vitro* culture. All plants used in this study were identified as *A. lancea* based on morphological characteristics by the first author.

### 2.2. ***De novo*** genome assembly

Genome sequencing was performed using a single individual of *A. lancea* (accession Y-T16-69). High-molecular-weight genomic DNA was extracted from young leaf tissues using a Genomic-tips Kit (Qiagen, Hilden, Germany) according to the manufacturer's instructions.

For short-read sequencing, PCR-free libraries were prepared using the TruSeq DNA PCR-Free LT Sample Prep Kit (Illumina, San Diego, CA, USA). Sequencing was performed using an Illumina HiSeq X platform to generate paired-end reads with a read length of 150 bp. For long-read sequencing, HiFi reads were obtained by constructing a library using the SMRTbell Prep Kit 3.0 (Pacific Biosciences, Menlo Park, CA, USA). Sequencing was performed using the PacBio Revio System. For optical genome mapping (OGM), ultra-high-molecular-weight (UHMW) DNA was isolated using the Bionano Prep Plant Tissue DNA Isolation Kit (Bionano Genomics, San Diego, CA, USA, Cat No. 80003). Fluorescent labeling of the isolated UHMW DNA (750 ng) was performed using a Direct Label and Stain (DLS) Kit (Bionano Genomics, Cat No. 80005) in accordance with the Bionano Prep Direct Label and Stain (DLS) protocol (Bionano Document No. 30206, Revision G). In brief, UHMW DNA molecules were labeled with the DL-green fluorophore via Direct Label Enzyme (DLE-1) to generate sequence-specific patterns. DNA backbones were stained overnight with a fluorescent dye to visualize the full-length DNA molecules. Labeled UHMW DNA was loaded onto a Saphyr Chip G1.2 (Bionano Genomics, Cat No. 20319), and molecular imaging was performed using the Bionano Saphyr platform. The Hi-C library was constructed using an Omni-C Kit (Dovetail Genomics, Scott Valley, CA, USA). The resulting Omni-C libraries were sequenced on a DNBSEQ-G400RS platform (MGI Tech Co., Ltd., Shenzhen, China) to generate paired-end reads with a length of 150 bp.

Short-read sequencing data were used to estimate genome size using Jellyfish.^19^ HiFi long reads were assembled using Hifiasm (version 0.16.1) ,^20^ resulting in phased assemblies that included a primary assembly and two haplotype-resolved assemblies (hap1 and hap2). Potential organelle sequences were excluded from the assembled genomes by conducting similarity searches against the organelle sequences of the Asteraceae relatives, *Helianthus annuus*, *Lactuca sativa*, and *Pulicaria dysenterica*: CM007908.2, CM049014.1, NC_007578.1, NC_042756.1, OX381670.1, and OX381671.1.

After removing the organellar sequences, the OGM molecules were aligned to the nuclear scaffolds to reconstruct the contigs and improve the assembly contiguity. *De novo* assembly and scaffolding were performed separately using the Bionano Solve software (v3.8.1) (Bionano Genomics) based on primary assembly, hap1, and hap2. Subsequently, OGM-based scaffolding was performed using YaHS v2.0.1^21^ to generate chromosome-scale scaffolds. The resulting primary, hap1, and hap2 scaffold assemblies were compared using RaGOO^22^ to identify large-scale structural inconsistencies. Scaffolds that were clearly indicative of assembly errors were manually corrected by rearranging or reconnecting the contig segments. Gene prediction was performed using Helixer^23^ and the predicted genes were functionally annotated using emapper (v2.1.12) implemented in eggNOG (v5.0).^24^ Repeat masking was not performed prior to Helixer-based gene prediction. Assembly completeness was assessed using BUSCO v5.5.0^25^ with the embryophyte_odb10 dataset. Repetitive sequences in the assembly were identified using RepeatMasker (v4.2.2) (https://www.repeatmasker.org), based on repeat sequences registered in Repbase (https://www.girinst.org/repbase) and a de novo repeat library built with RepeatModeler (v2.0.7) (https://www.repeatmasker.org).

### 2.3. GC–MS-based phenotyping of essential oil compounds

The dried rhizomes were pulverized using a vibrating mill (TI-200, Cosmic Mechanical Technology). The four essential oil compounds, β-eudesmol, hinesol, atractylon, and atractylodin, were quantified using gas chromatography–mass spectrometry (GC–MS) as described previously.^10^ Briefly, powdered rhizome samples (0.5 g) were extracted twice with n-hexane. For GC-MS analysis, the combined extracts were spiked with phenanthrene as an internal standard and adjusted to a final volume of 50 mL using n-hexane. GC–MS analysis was performed using an Agilent 7890 gas chromatograph coupled with a 5975 mass-selective detector (Agilent Technologies) equipped with a DB-WAX capillary column (30 m × 250 μm i.d., 0.25 μm film thickness). Helium was used as the carrier gas at a flow rate of 1.0 mL min⁻^1^. The compounds were quantified on a dry-weight basis.

### 2.4. Genotyping data

Genomic DNA was extracted from 480 *A. lancea* lines, and single-nucleotide polymorphism (SNP) genotyping was performed using double-digest restriction-site-associated DNA sequencing (ddRAD-seq) according to the protocol described by Shirasawa *et al*.^26^ ddRAD-seq libraries were prepared using the restriction enzymes *PstI* and *MspI*, and sequencing was conducted on the DNBSEQ-G400RS platform. Raw sequence reads were mapped to the primary genome sequences constructed in this study using Bowtie2.^27^ Variant calling was performed using SAMtools^28^ according to the procedure described by Shirasawa *et al*.^26^ SNPs were filtered using VCFtools^29^ (v0.1.16) based on the following criteria: removal of indels and multi-allelic variants; minimum read depth of 10; maximum read depth of 200; genotype quality (GQ) > 10; exclusion of individuals with a missing genotype rate greater than 0.3; a maximum SNP missing rate of 0.7; and a minor allele frequency (MAF) ≥ 0.05. As a result of individual-level filtering based on missing genotype rates, 18 lines were excluded, and 462 lines were retained for downstream analyses. Missing genotypes were imputed using Beagle^30^ version 5.5, and 29,136 SNPs were retained for the genome-wide association studies (GWAS) and genomic prediction analyses.

For linkage disequilibrium (LD) decay analysis, SNPs were filtered using similar criteria, except that a more permissive missing rate threshold (≤ 0.9) was applied and genotype imputation was omitted, because LD estimation is sensitive to imputed genotypes. Pairwise LD between SNPs was estimated using the squared correlation coefficient (*r*^2^) with VCFtools v0.1.16.^29^ LD was calculated for SNP pairs located within 1 Mb of the same chromosome. LD decay was visualized as a function of physical distance by smoothing *r*^2^ values using local polynomial regression with kernel weighting, implemented with the locpoly function in the R package KernSmooth.^31^ LD decay curves were plotted for marker pairs within 500 kb to illustrate the decay of LD with increasing physical distance.

### 2.5. Genomic heritability

Genomic heritability was estimated using a linear mixed model in the R package rrBLUP.^32^ An additive genomic relationship matrix was constructed from SNP genotypes coded as 0, 1, and 2 using the A. mat function. Prior to analysis, the genotype and phenotype datasets were strictly matched by line identity, and the phenotypes were reordered to correspond exactly to the genotype matrix. The model y=μ+g+e was fitted, where genomic effects were assumed to follow $g\;∼\;N\;(0,\;\;\sigma_{g}^{2}\;K)$ and residuals $e\;∼\;N\;(0,\;\;\sigma_{e}^{2}\;I)$. Genomic heritability was calculated as $h^{2}=\sigma_{g}^{2}\;/\;(\sigma_{g}^{2}\;+\;\sigma_{e}^{2})$ using variance components estimated by restricted maximum likelihood (REML).

### 2.6. GWAS

Prior to the GWAS, phenotypic distributions were examined for each trait. Because several traits exhibited strong right skewness, phenotypes were transformed to improve residual normality and homogeneity of variance. Log-transformation was applied to atractylodin, hinesol, and β-eudesmol levels, whereas rank-based inverse normal transformation (INT) was applied to atractylon levels. The GWAS was performed using the SNPs that passed the quality control and imputation procedures described above. The genotype and phenotype data were matched by line identity. The GWAS was conducted using a linear mixed model (LMM) implemented in the GWAS function of the R package rrBLUP.^32^ A marker-based additive genomic relationship matrix (GRM) was constructed from the filtered and Beagle-imputed genotype matrix using the A. mat function. To account for the population structure and genetic relatedness among lines, the GRM was incorporated as a random effect, and the top three principal components derived from the genome-wide marker data were included as covariates (n.PC = 3). Statistical significance was assessed using *P*-values obtained for each SNP, and multiple testing corrections were performed using the Benjamini–Hochberg false discovery rate (FDR). SNPs with FDR < 0.05 were considered significant. Manhattan and quantile–quantile (*Q–Q*) plots were generated using the R package qqman^33^ to visualize genome-wide association signals and assess the distribution of test statistics.

### 2.7. Genomic prediction

Genomic prediction analyses are conducted using raw phenotypic values to allow direct interpretation of the prediction results on the original trait scale. To evaluate the accuracy of genomic prediction of essential oil component levels, several regression-based and machine learning models were applied. Penalized linear regression models, including ridge regression (*α* = 0), LASSO (*α* = 1), and elastic net (*α* = 0.5), were implemented using the R package glmnet.^34^ Genomic best linear unbiased prediction (GBLUP) with ridge regression (RR) and Gaussian kernels was performed using the kinship.BLUP function of the R package rrBLUP,^32^ with variance components estimated by REML. In addition, Bayesian regression models (BayesA, BayesB, and BayesC) were evaluated using the R package BGLR,^35^ and a nonlinear machine learning approach, random forest, was implemented using the R package Rborist.^36^ The prediction accuracy was assessed using repeated cross-validation. Specifically, a 10-fold cross-validation was conducted and repeated ten times with different random partitions of the data. In each replicate, the population was randomly divided into ten subsets, one of which was used as the validation set, and the remaining nine subsets served as the reference population for model training. This procedure was repeated until all the individuals were predicted once in each replicate. Prediction accuracy (*r*) was calculated as the Pearson correlation coefficient between the observed and predicted genotypic values across all individuals.

To explore the distribution of marker effects across the genome, marker effects were evaluated using representative genomic prediction models. Specifically, marker effects were estimated using GBLUP-RR and BayesC implemented in R packages rrBLUP and BGLR, respectively.^32,35^ In the GBLUP-RR model, marker effects were assumed to follow a common normal distribution, whereas BayesC allowed a subset of markers to have nonzero effects. The filtered and Beagle-imputed genotype matrix was converted to an additive genotype matrix and standardized before model fitting. For GBLUP-RR model, marker effects were estimated using the mixed.solve function in rrBLUP, and the fitted random marker effects (*u*) were extracted as SNP effects. For BayesC, marker effects were estimated using the BGLR function with the BayesC model, and posterior mean estimates of marker effects were extracted from the fitted model. For both models, the absolute values of the estimated marker effects were visualized along the genome based on the physical positions of SNPs on each chromosome using Manhattan-style plots generated with the R package qqman.^33^

## 3. Results

### 3.1. De novo genome assembly of *A. lancea*

Through k-mer analysis of short-read sequencing data, the genome size of *A. lance*a was estimated to be approximately 4.3 Gb. The k-mer distribution revealed a pronounced heterozygous peak, indicating a high level of heterozygosity (Figure S1). A *de novo* genome assembly of *A. lancea* was generated to serve as a reference for subsequent genomic analyses (Table S2). The primary assembly comprised 1,077 scaffolds with a total length of 4.79 Gb (Table 1). Among these, 12 contigs corresponding to chromosome-scale sequences accounted for 4.45 Gb of the assembly, whereas the remaining 1,065 unplaced scaffolds had a total length of 342 Mb. The length of scaffold N50 of the primary assembly was 337.9 Mb, with the longest scaffold being 610.5 Mb. Genome completeness, assessed using BUSCO v5.5.0, indicated 98.9% completeness of the primary assembly, with 98.5% of the complete BUSCOs located on chromosome-scale sequences (Table 1), supporting the high completeness of the assembled genome. Repetitive sequences accounted for 4.0 Gb (82.9%) of the assembly, comprising LTR elements (2.4 Gb: 51.1%) followed by DNA transposons (298.3 Mb: 6.2%). Repeat sequences unavailable in public databases totaled 1.0 Gb (21.2%) (Table S3). The primary assembly was used as the reference genome for downstream analyses, including SNP calling, GWAS, and genomic prediction.

**Table 1.** Summary statistics of the *Atractylodes lancea* genome assembly (primary).

Statistics	Primary assembly	Chromosomes	Unplaced scaffolds
Number of sequences	1,077	12	1,065
Total length (bp)	4,791,082,237	4,448,898,171	342,184,066
Average length (bp)	4,448,544	370,741,514	321,300
Max length (bp)	610,539,435	610,539,435	10,347,447
Min length (bp)	10,968	260,554,996	10,968
N50 length (bp)	337,946,156	337,946,156	1,480,758
BUSCO completeness	98.9% [S:85.8%,D:13.1%],F:0.6%,M:0.5%,*n*:1614	98.5% [S:86.7%,D:11.8%],F:0.6%,M:0.9%,*n*:1614	2.8% [S:2.7%,D:0.1%],F:0.9%,M:96.3%,*n*:1614
			

BUSCO categories are defined as follows: S, complete and single-copy BUSCOs; D, complete and duplicated BUSCOs; F, fragmented BUSCOs; M, missing BUSCOs; and *n*, total number of BUSCO groups searched.

In addition, Helixer-based structural annotation identified 90,695 predicted gene models in the primary assembly. BUSCO assessment of the Helixer gene predictions using the embryophyta_odb10 dataset indicated 93.9% completeness. Detailed annotation statistics for the primary and haplotype-resolved assemblies are provided in Table S4. Functional annotation using eggNOG-mapper assigned at least one functional annotation to 63,048 (69.5%) of the 90,695 Helixer-predicted gene models, with orthologous matches identified for 65,437 gene models (72.2%). Detailed functional annotation statistics are provided in Table S5. Additionally, putative terpene synthase and cytochrome P450 gene models identified based on eggNOG-mapper annotations are listed in Tables S6 and S7, respectively. A total of 25 putative terpene synthase and 508 putative cytochrome P450 gene models were identified in the primary assembly.

### 3.2. Phenotypic variation and genomic heritability of essential oil compound levels

To assess phenotypic variation in the levels of essential oil components for GWAS and genomic prediction, we quantified four metabolites, atractylodin, hinesol, β-eudesmol, and atractylon, in rhizomes from 480 lines of *A. lancea*. All four compounds exhibited wide variations across the population (Table 2), supporting their suitability for subsequent GWAS and genomic prediction analyses. Pairwise correlations among the four essential oil compound levels were examined to evaluate relationships among breeding targets (Figure S2). Atractylon showed a moderate positive correlation with atractylodin (*r* = 0.59), and hinesol showed a weak-to-moderate positive correlation with β-eudesmol (*r* = 0.40), whereas the other trait pairs showed little or no correlation.

**Table 2.** Descriptive statistics of essential oil compound levels in *Atractylodes lancea*.

Essential oil compound	*n*	Mean mg/g	SD	Min mg/g	Max mg/g	Range mg/g
Atractylodin	480	2.2	1.3	0.2	8.1	7.9
Hinesol	480	20.6	10.5	1.7	83.8	82.1
β-Eudesmol	480	18.0	7.6	2.7	45.0	42.3
Atractylon	480	1.0	2.4	0.02	20.2	20.2

Summary statistics of four essential oil compounds, atractylodin, hinesol, β-eudesmol, and atractylon, measured in rhizomes of 480 *A. lancea* lines. Trait values are expressed in mg g⁻^1^ dry weight.

**Fig. 1.**
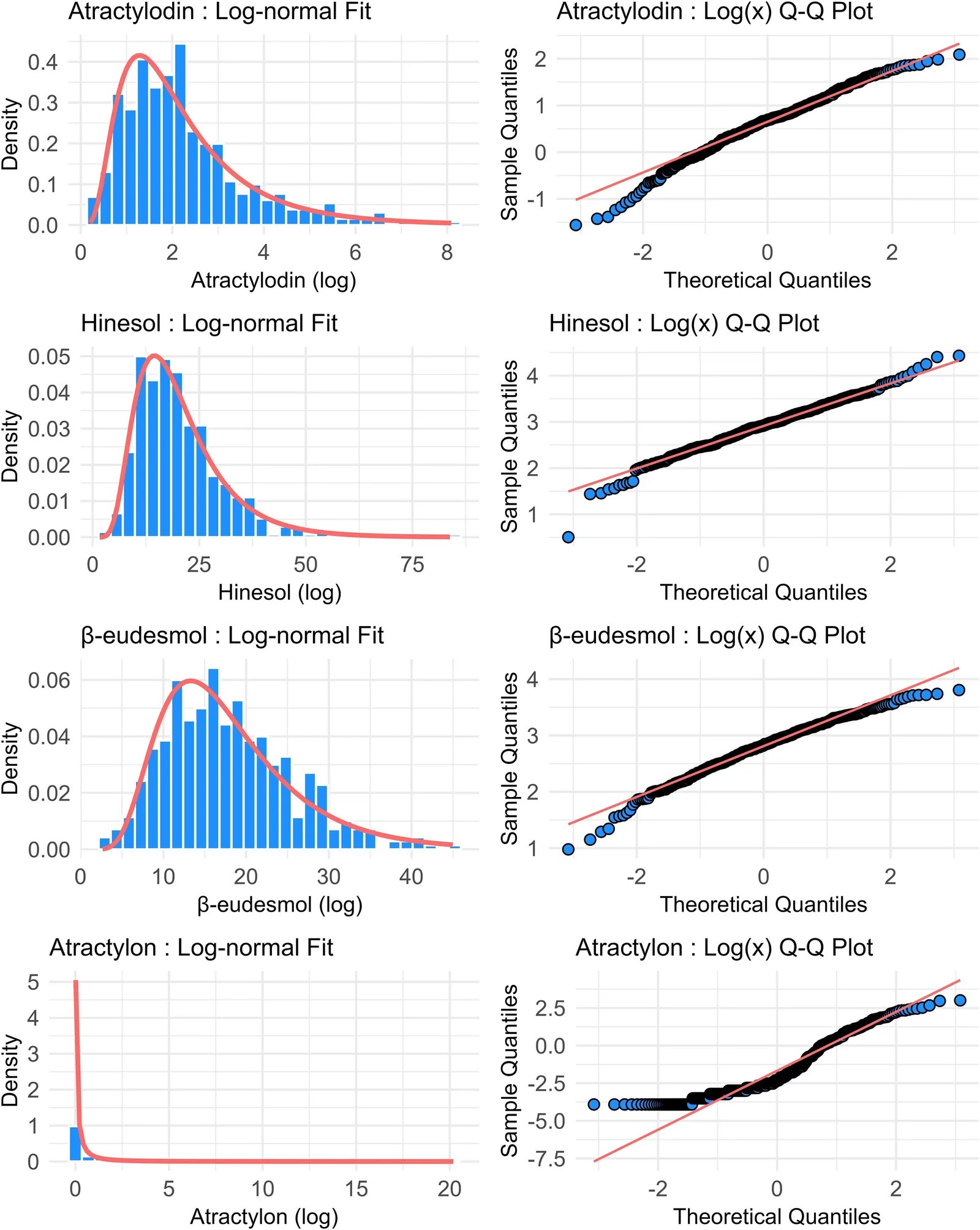
Phenotypic distributions of log-transformed essential oil compound levels in *Atractylodes lancea*. Histograms overlaid with fitted normal density curves (left column) and corresponding quantile–quantile (*Q–Q*) plots (right column) are shown for log-transformed values of atractylodin, hinesol, β-eudesmol, and atractylon measured in rhizomes of 480 *A. lancea* lines.

Normality of the phenotypic distributions was assessed using histograms, quantile–quantile plots, and the Shapiro–Wilk test. The raw phenotypic values for all four compounds deviated substantially from normality (Figure S3). Among them, atractylon showed a particularly skewed distribution, with a minimum observed value of 0.02 mg/g and many low-value observations (Table 2, Figure S3). Log transformation markedly improved the distributions of atractylodin, hinesol, and β-eudesmol, resulting in distributions that closely approximated normality (Fig. 1). However, atractylon remained abnormal, even after log transformation (Fig. 1). Therefore, inverse normal transformation (INT) was applied to atractylon, which substantially improved its distributional normality (Figure S4). Since the models used for GWAS and genomic heritability estimation assume normally distributed residuals, log-transformed values were used for atractylodin, hinesol, and β-eudesmol, whereas INT-transformed values were used for atractylon in these analyses. In contrast, genomic prediction analyses were performed using untransformed phenotypic values, because the absolute levels of essential oil compounds are crucial for breeding applications.

Genomic heritability (*h*^2^) was estimated for each trait to evaluate the genetic contribution to the variation in the essential oil compound levels. Genomic heritability was high for all essential oil compounds, ranging from 0.758 to 0.915 (Table 3). The highest genomic heritability was observed for hinesol (*h*^2^ = 0.915), followed by β-eudesmol (*h*^2^ = 0.877) and atractylodin (*h*^2^ = 0.875), whereas atractylon showed a relatively lower but still substantial genomic heritability (*h*^2^ = 0.758).

**Table 3.** Genomic heritability (*h*^2^) of essential oil compound levels estimated using a linear mixed model in *Atractylodes lancea*.

Trait (essential oil compound)	Genomic heritability (*h*^2^)
Atractylodin	0.875
Hinesol	0.915
β-Eudesmol	0.877
Atractylon	0.758

Genomic heritability (*h*^2^) was estimated using SNP-based genomic relationship matrices.

### 3.3. GWAS for essential oil compound levels

Prior to genome-wide association analysis, genome-wide linkage disequilibrium (LD) decay was evaluated (Figure S5). LD declined rapidly with physical distance, with the average *r*^2^ decreasing to 0.2 at approximately 14.0 kb and to 0.1 at approximately 74.1 kb. In addition, the mean *r*^2^ between adjacent SNPs was 0.23, suggesting that the marker set retained useful genome-wide information for genomic prediction.

**Fig. 2.**
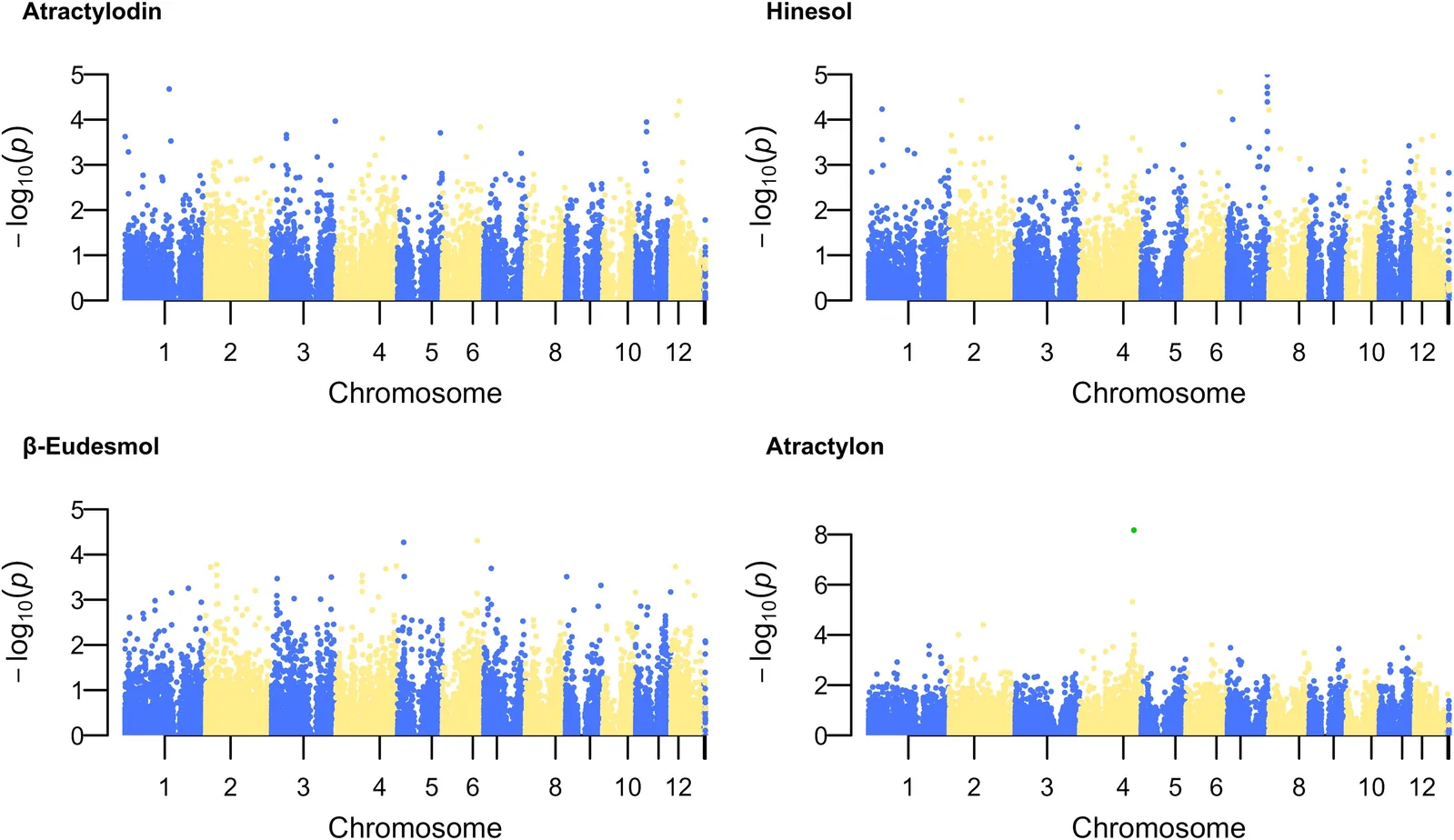
Genome-wide association results for essential oil compound levels in *Atractylodes lancea*. Manhattan plots showing genome-wide association results for atractylodin, hinesol, β-eudesmol, and atractylon. The x-axis represents genomic locations across chromosomes, and the y-axis indicates −log_10_(p) values for SNP–trait associations. SNPs exceeding the false discovery rate (FDR) threshold of 5% are highlighted in green.

To account for the population structure, principal component analysis (PCA) was performed using genome-wide SNP data (Figure S6). The first principal component (PC1) explained 65.82% of the total genetic variation, whereas PC2 and PC3 explained 2.47% and 1.04% of it, respectively. Based on these results, GWAS included the first three principal components as covariates. GWAS were conducted separately for atractylodin, hinesol, β-eudesmol, and atractylon to identify genomic regions associated with variation in essential oil compound levels using genome-wide SNP markers. No significant SNPs associated with atractylodin, hinesol, and β-eudesmol were detected at a false discovery rate (FDR) threshold of 5% (Fig. 2). In contrast, a single SNP that was significantly associated with atractylon was detected at the same significance threshold (Fig. 2). Quantile–quantile (*Q–Q*) plots indicated no substantial inflation of test statistics for any of the traits, suggesting that the population structure was adequately controlled in the GWAS models (Figure S7). To further evaluate the effect of the SNP significantly associated with atractylon, the lines were classified according to genotype (0, 1, or 2), and differences in atractylon level were assessed (Figure S8). Because inverse-normal transformed values were used in the GWAS, we examined both the original atractylon content and the inverse-normal transformed values. The original atractylon content was evaluated using the Kruskal–Wallis test, whereas the inverse-normal transformed values were evaluated using one-way ANOVA. Although variation in atractylon level was observed among the genotypic classes, no significant differences were detected among the genotypes at the 5% significance level for either the original atractylon content, based on the Kruskal–Wallis test, or the inverse-normal transformed values, based on one-way ANOVA.

### 3.4. Genomic prediction for essential oil compound levels

**Fig. 3.**
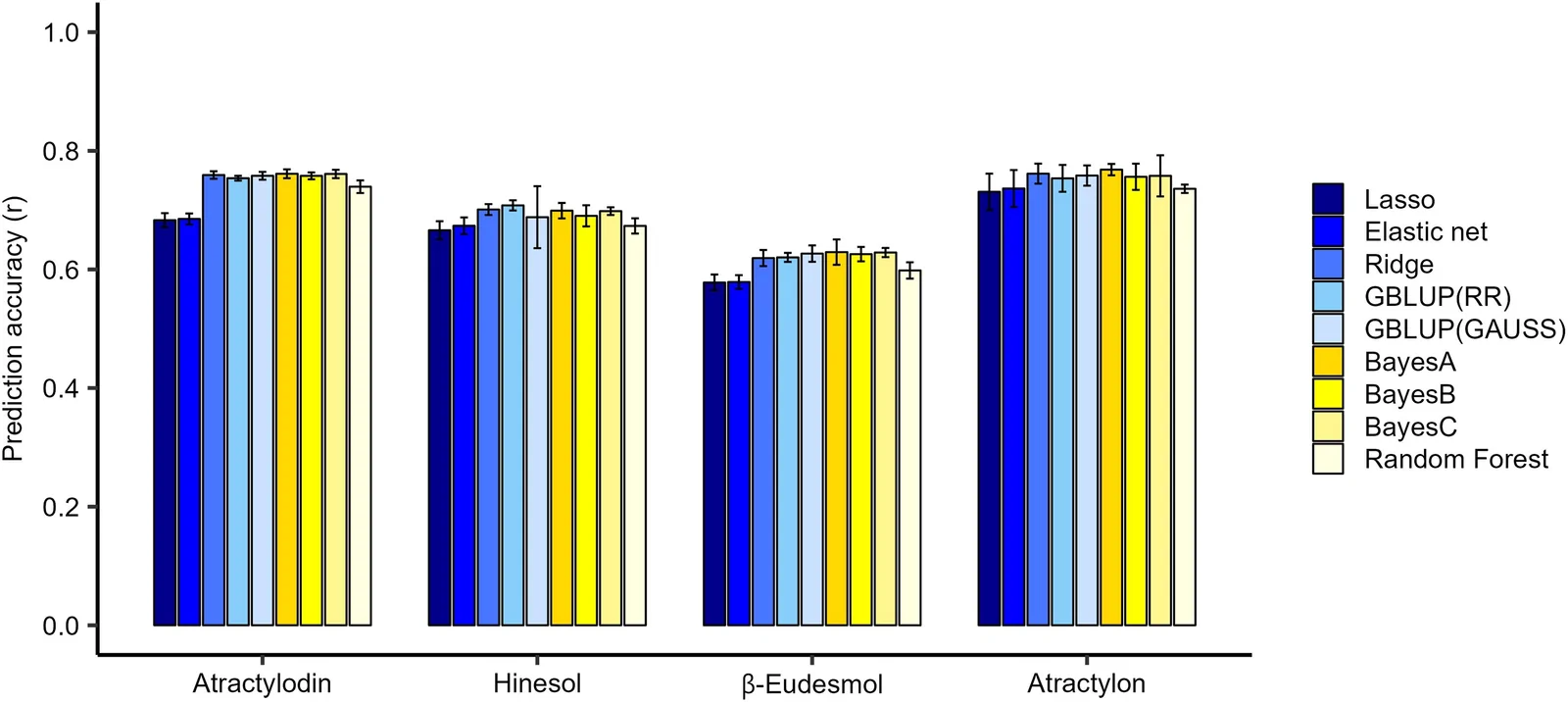
Genomic prediction accuracy for essential oil compound levels in *Atractylodes lancea*. The accuracy of predictions was evaluated using the Pearson correlation coefficient between the observed and predicted genotypic values derived from cross-validation. Genomic predictions were performed for the four essential oil compounds using nine prediction models: ridge regression, Lasso, Elastic Net, GBLUP-based models, Bayesian models (Bayes A, Bayes B, and Bayes C), and Random Forest. Bars indicate mean prediction accuracies across ten repeated cross-validation runs (*n* = 10), and error bars represent standard deviations.

Genomic prediction accuracy varied among the traits and prediction models (Fig. 3). The prediction accuracies were consistently high for atractylodin and atractylon, with mean prediction correlations generally exceeding 0.75 across the ridge regression, GBLUP-based, and Bayesian models. Although prediction accuracies for β-eudesmol and hinesol were lower than those for atractylodin and atractylon, prediction correlations for these compounds consistently exceeded 0.6 across most models, indicating reasonably high predictive performance. Clear differences in prediction performance were observed among the models. Ridge regression, GBLUP-based models, and Bayesian models (BayesA, BayesB, and BayesC) generally achieved comparable and high prediction accuracies for all four compounds. In contrast, Lasso and Elastic Net, which impose strong sparsity on marker effects, tended to show lower prediction accuracy, whereas the Random Forest model exhibited intermediate performance. The relative rankings of the prediction models were highly consistent across the compounds. The prediction accuracies remained stable across the cross-validation replicates for all models, exhibiting relatively small standard deviations. Overall, these findings indicate that the genomic prediction of essential oil compound levels in the rhizomes of *A. lancea* can achieve moderate-to-high accuracy across different compounds, with particularly strong performance for atractylodin and atractylon.

To evaluate whether SNPs with significant effects were detected by the genomic prediction models, SNP effect estimates were examined using the GBLUP (RR) and BayesC models (Figures S9 and S10). The GBLUP (RR) model assumes normally distributed marker effects with homogeneous variance across all SNPs, whereas the BayesC model allows for a proportion of SNPs to have zero effects. Nevertheless, in both models, the SNP effects were broadly distributed across the genome, and no SNPs with exceptionally large effects were observed for any of the four compounds. These results are consistent with the fact that the accuracy of genomic prediction is affected by the cumulative contribution of numerous small-effect loci.

## 4. Discussion

A high-quality reference genome is essential for reliable genome-wide analysis, including GWAS and genomic prediction. In this study, we performed the de novo genome assembly of *A. lancea* using PacBio HiFi long-read sequencing combined with optical genome mapping and Hi-C scaffolding. *A. lancea* has a basic chromosome number of 12,^37^ and in the present assembly, 12 chromosome-scale scaffolds accounted for approximately 92.9% of the total assembled genome length (4.45 Gb out of 4.79 Gb) (Table 1), indicating that the majority of the genomic sequences were successfully anchored to pseudochromosomes. The present assembly had a scaffold N50 of 337.9 Mb, and BUSCO analysis revealed a completeness of 98.5% for the chromosome-scale sequences (Table 1), demonstrating that the most conserved single-copy orthologs were retained. The repeat annotation revealed that the *A. lancea* genome is highly repeat-rich (Table S3), indicating substantial genomic complexity. The primary assembly was approximately 11% larger than the genome size estimated from k-mer analysis (Figure S1 and Table 1). The repeat-rich genomic architecture likely increased assembly complexity and may have contributed to the difference between the k-mer based genome size estimate and the assembly size. Similar discrepancies have been reported in other organisms, depending on species and estimation methods.^38,39^ In addition, the pronounced heterozygous peak in the k-mer distribution suggests that some allelic or haplotypic regions were retained as separate sequences in the primary assembly rather than being fully collapsed. The retention of these regions as separate sequences may also have increased the assembled genome size. This interpretation is consistent with the duplicated BUSCO proportion of 13.1%, suggesting that a subset of conserved genes may be represented more than once because of residual haplotypic duplication.

Previous chromosome-scale genome assemblies of *A. lancea* have been reported by Zhang *et al*. and Zhu *et al*.^15,16^ Zhang *et al.* reported a 4.01 Gb assembly with a scaffold N50 of 289.33 Mb, 12 pseudochromosomes, and BUSCO completeness of 89.90%.^15^ Zhu *et al*. reported a 4.24 Gb chromosome-level assembly with a scaffold N50 of 435.30 Mb, and BUSCO completeness of 95.4%.^16^ Although the assembled genome sequences from these studies are not directly accessible through public repositories, the reported assembly statistics are broadly comparable to those obtained in the present study. These comparisons indicate that the present assembly has chromosome-scale contiguity comparable to previously reported assemblies and high gene-space completeness, supporting its suitability as a reference genome for downstream genome-wide analyses. An important feature of the present assembly is that, to the best of our knowledge, it is the first chromosome-scale *A. lancea* genome assembly that is directly accessible through public repositories without access restrictions. Based on this chromosome-scale assembly, the reference genome generated in the present study was used for all subsequent genome-wide analyses, including genomic heritability, linkage disequilibrium estimation, GWAS, and genomic prediction.

First, we estimated the genomic heritability of essential oil compound levels in the *A. lancea* population. The genomic heritability of essential oil compounds was high (Table 3), indicating that a large proportion of the phenotypic variation can be explained by genetic effects. In our previous study, the broad-sense heritability of β-eudesmol, hinesol, atractylon, and atractylodin levels was estimated using clonal lines and variance component analysis.^10^ The estimated broad-sense heritability values for these essential oil compounds were high, ranging from 0.77 to 0.87. These results are consistent with the present findings and demonstrate that genetic factors contribute substantially to variations in essential oil compound levels. Although the approaches used to estimate heritability differed between the two studies, the overall conclusions were consistent, supporting the robustness of genetic control over essential oil compound levels in *A. lancea*. Because only a single biological replicate was used for each line in the present study, within-line variability could not be directly estimated. To assess the possible magnitude of such variability, we reanalyzed the publicly available individual-level data from the previous study that used clonal lines of *A. lancea*.^10^ This analysis showed median within-line coefficients of variation of 12.5%, 13.6%, 13.7%, and 9.6% for β-eudesmol, hinesol, atractylon, and atractylodin, respectively. These values reflect variation among individual plants within a line and may include biological variation, micro-environmental effects, and analytical variation associated with sample preparation and GC-MS quantification. Therefore, phenotypic values in the present study likely include comparable non-genetic variation, which may affect genomic heritability estimates in either direction and may reduce or obscure the interpretation of genomic prediction accuracy as purely genomic signal. Thus, the heritability estimates and prediction accuracies reported here should be interpreted with caution. Despite this limitation, the consistently high heritability observed in the present and previous studies supports a substantial genetic contribution to essential oil compound variation in *A. lancea*. Genome-wide single nucleotide polymorphism (SNP)-based analyses in other plant species have shown that specialized metabolites often exhibit moderate to high heritability. For example, in oat, genomic heritability estimates for metabolite traits indicated that specialized metabolites tended to exhibit higher heritability than primary metabolites.^40^ In non-model plant species, studies estimating genomic heritability remain limited; however, relatively high genomic heritability (*h*^2^ = 0.5 to 0.8) has been reported for specialized metabolite levels, such as volatile organic compounds, in blueberry.^41^ In the present study, genomic heritability of essential oil compound levels in *A. lancea* was comparable to or higher than that reported previously. Based on this consistent evidence of genetic influence, we conducted genome-wide association analyses to examine whether genomic regions with large contributions to essential oil compound level could be identified.

However, despite the high genomic heritability of essential oil compound levels, genome-wide association analyses did not detect SNPs that reached significance at an FDR threshold of 5% for atractylodin, β-eudesmol, and hinesol levels (Fig. 2), indicating limited power to identify individual loci with large effects. Although a single SNP associated with atractylon level was identified (Fig. 2), genotype-based comparisons did not reveal significant differences in atractylon levels (Figure S8), suggesting that the variation in atractylon level is unlikely to be controlled by a single locus with a large effect. Although genomic heritability is high, the absence of significant GWAS signals with large effects suggests that the trait is controlled by many loci with small individual effects.^42^ Several factors may have limited the power of GWAS in the present population. First, the PCA revealed strong genetic structure among the retained *A. lancea* lines, with PC1 explaining 65.82% of the total genetic variation and four apparent clusters observed in the PC1–PC2 space (Figure S6). The lines were derived from seed-derived progeny obtained under natural breeding conditions from source materials that included clonal lines of different origins characterized in previous studies.^12^ Given the reproductive characteristics of *A. lancea,* in which seed production is mainly associated with female plants, the effective number of seed parents may have been limited under natural breeding conditions. Thus, the observed clusters may reflect genetic differentiation among source lineages or family structure retained in the derived population. No additional samples were removed as PCA outliers after genotype quality control, and population structure was accounted for in GWAS by including principal components and a kinship matrix. Nevertheless, such strong structure may have reduced the effective population size and thereby limited the power to detect loci with small effects.^43^ Second, marker density may have limited the resolution and power of genome-wide analyses. LD patterns provide important insights into the resolution and power of genome-wide analyses.^44^ In the present *A. lancea* population, LD decayed to r^2^ = 0.2 within approximately 14 kb and further to *r*^2^ = 0.1 within approximately 74 kb (Figure S5). Considering the large genome size of *A. lancea* (approximately 4.79 Gb), the use of 29,136 SNPs corresponded to an average marker density of approximately one SNP per 164 kb across the genome. Therefore, many genomic regions were likely not sufficiently tagged by the available markers, which may have reduced the power and resolution of GWAS. In future studies, the chromosome-scale reference genome generated in this study could help address the limitation of low marker density. For example, representative lines from each genetic cluster could be resequenced to construct a high-density SNP catalogue and haplotype reference panel. Larger breeding populations could then be genotyped by low-coverage whole-genome sequencing or skim sequencing, followed by genotype imputation, an approach that has been used to generate cost-effective, high-density genome-wide marker datasets in crop species.^45^ This approach would provide denser genome-wide markers at lower cost than high-depth resequencing of all lines and would be particularly useful for *A. lancea*, given its large genome size and rapid LD decay. Denser marker coverage would improve genome-wide tagging and may also contribute to higher GWAS resolution. This limited marker coverage may also have affected genomic prediction, because prediction accuracy depends partly on the extent to which markers capture LD with causal variants. In contrast to GWAS, however, genomic prediction may remain effective even when individual marker–trait associations are difficult to detect, because it uses genome-wide marker information to predict genetic values.^46,47^ In addition, previous studies have shown that accurate genomic prediction can be achieved for high-heritability traits (*h*^2^ ≈ 0.5) even when the average *r*^2^ between adjacent markers is approximately 0.10–0.14.^48^ The mean *r*^2^ between adjacent SNPs was 0.23 in the present study, which is comparable to or slightly higher than the *r*^2^ = 0.20 level often used as a benchmark for adjacent-marker LD in genomic selection.^49^ Therefore, although denser marker coverage would likely improve genome-wide tagging, the marker set in the present study likely retained useful genome-wide predictive information.

Based on these considerations, we evaluated the predictive performance of the genomic prediction models for essential oil compound levels. Genomic prediction was examined using nine machine learning models, and the prediction accuracy was evaluated using 10-fold cross-validation. A high prediction accuracy was observed across multiple models for essential oil compound levels (Fig. 3). Among the evaluated models, ridge regression and GBLUP consistently showed higher prediction accuracy than LASSO and elastic net for all four essential oil compounds (Fig. 3). Ridge regression and GBLUP-based models assume genome-wide contributions from many markers with small effects and apply dense shrinkage, whereas LASSO and elastic net enforce sparsity by selecting a limited number of markers with nonzero effects.^32,50–52^ The superior performance of ridge regression and GBLUP-based models, together with the intermediate performance of elastic net compared to LASSO, suggests that essential oil compound levels in *A. lancea* are likely polygenic traits. Bayesian models (BayesA, BayesB, and BayesC) also showed high prediction accuracy, similar to the ridge regression and GBLUP-based models (Fig. 3). In principle, Bayesian models allow marker-specific shrinkage and variable selection.^53^ However, when a trait is influenced by many loci with small effects, the prediction accuracy is comparable to that of ridge regression and GBLUP-based models.^54,55^ Furthermore, marker effect estimates from GBLUP and BayesC did not identify SNPs with exceptionally large effects, similar to the results of the GWAS analysis (Figures S9 and S10). This observation further supports the conclusion that essential oil compound levels in *A. lancea* are governed by a polygenic genetic architecture.

GWAS and genomic predictions have been applied to medicinal plants and other perennial and woody species with long growth cycles to shorten breeding periods and improve breeding efficiency. In citrus and Japanese pear, GWAS and genomic prediction have been applied to fruit quality traits, such as sugar content, fruit size, and peel color, and these studies suggested that the combined use of GWAS and genomic selection is important for effective breeding.^56,57^ In blueberry, GWAS and genomic prediction have been applied to volatile compounds, showing that some volatiles are suitable for marker-assisted selection, whereas others are better predicted using genomic prediction because of their polygenic architecture.^41^ Similarly, in tea, GWAS and genomic prediction have been conducted for specialized metabolites, such as catechins and caffeine. Although clear major GWAS peaks were not detected, moderate prediction accuracy was achieved using genomic prediction.^58^ These results are similar to those observed for essential oil compounds in *A. lancea*. Although specialized metabolites can exhibit diverse genetic architectures depending on the plant species and metabolite type, the essential oil compounds in *A. lancea* are likely to be predominantly polygenic.

Essential oil compounds in *A. lancea*, including β-eudesmol, hinesol, and atractylodin, are pharmacologically important constituents. β-Eudesmol and hinesol exhibit anti-ulcer activity, whereas atractylodin can ameliorate delayed gastric emptying.^5,6^ For these reasons, these compounds are thought to play an important role in traditional medicine prescriptions containing *A. lancea*. However, cultivated *A. lancea* have been reported to exhibit lower levels of essential oil compounds than wild accessions, posing a major challenge for cultivation and breeding.^9^ In this study, we demonstrated the potential of breeding for pharmacologically active compounds in *A. lancea* using genomic prediction. These results indicated that genomic prediction can be used as a breeding tool for medicinal plants. In recent years, genomic selection has been applied to specialized metabolites in perilla, a medicinal plant, suggesting the potential for efficient genetic improvement.^59,60^ Furthermore, genomic selection was found to be effective for improving polygenic traits in allogamous plants such as common buckwheat.^61^ These findings suggest that efficient breeding of *A. lancea*, an allogamous plant, may be achieved through genomic selection. In practical breeding programs, the observed correlations among essential oil compounds should also be considered when designing selection strategies. In the present study, moderate positive correlations were observed between hinesol and β-eudesmol and between atractylon and atractylodin (Figure S2). The correlation between hinesol and β-eudesmol is consistent with their structural similarity and previous observations, whereas the correlation between atractylon and atractylodin should be interpreted cautiously because these compounds are structurally less similar and a similar relationship was not observed in previous studies.^10^ These results suggest that simultaneous improvement may be feasible for positively correlated compounds, whereas compounds showing weak or no correlations may require independent selection strategies.

In conclusion, we generated and released a chromosome-scale reference genome for *A. lancea* to establish a genomic foundation for genome-assisted breeding. Using this resource, the potential of genome-assisted breeding for essential oil compound levels in *A. lancea* was evaluated using GWAS and genomic prediction analysis. Although the GWAS did not identify loci with large effects, the results of genomic prediction indicated that essential oil compound levels are likely controlled by a polygenic genetic architecture and can be predicted with high accuracy using genome-wide marker information. These findings suggest that genomic selection is a promising strategy for efficient breeding of pharmacologically important traits in *A. lancea*, an allogamous medicinal plant with a long cultivation period.

## Supplementary Material

dsag014_Supplementary_Data

## Data Availability

Sequence reads are available from the DNA Data Bank of Japan (DDBJ) Sequence Read Archive (DRA) under the Bio Project number PRJDB40368. The assembled scaffold sequences, gene sequences, and annotation files are available from the Kazusa Genome Atlas **(**https://genome.kazusa.or.jp/home/) and PlantGARDEN (https://plantgarden.jp/).
