# Supplementary material for: Chromosome-scale genome assembly and genomic prediction of essential oil compounds in Atractylodes lancea for genomics-assisted breeding
Source: DNA Res. 2026 Sep 10;33(5):dsag014. doi: 10.1093/dnares/dsag014 (PMC13601803; doi:10.1093/dnares/dsag014)
Supplement: Supplementary file 1 [file dsag014_supplementary_data.zip › Supplementary Figures_Revise_DNAR-2026-016.docx]

**Supplementary Fig. 1.** K-mer frequency distribution of short-read data from *Atractylodes lancea*, indicating an estimated genome size of approximately 4.3 Gb and high heterozygosity.

**Supplementary Fig. 2.** Pairwise correlations among essential oil compounds in *A. lancea*. Pearson’s correlation coefficients were calculated for all pairwise combinations of the four essential oil compounds. Blue and red circles indicate positive and negative correlations, respectively, and circle size reflects the strength of the correlation. Correlation coefficients are shown in each cell.

**Supplementary Fig. 3. Phenotypic distributions of raw essential oil compound levels in *A. lancea*.**
Histograms overlaid with fitted normal density curves (left column) and corresponding quantile–quantile (Q–Q) plots (right column) are shown for raw values of atractylodin, hinesol, β-eudesmol, and atractylon measured in rhizomes of 480 *A. lancea* lines. These distributions illustrate the deviation from normality prior to log transformation, as shown in Fig. 1.

**Supplementary Fig. 4.** Histogram and quantile–quantile plot of atractylon after inverse normal transformation (INT), showing improved normality compared with raw and log-transformed values.

**Supplementary Fig. 5.** Genome-wide linkage disequilibrium (LD) decay in *A. lancea*
Pairwise LD (r²) between SNPs was calculated as a function of physical distance using genome-wide marker pairs. The solid line represents the smoothed LD decay curve. LD declined rapidly with increasing distance, reaching r² values of 0.2 and 0.1 at approximately 14 kb and 74 kb, respectively.

**Supplementary Fig. 6**. Principal component analysis of genome-wide SNP data in *A. lancea*.

Principal component analysis (PCA) was performed using genome-wide SNP data to assess population structure and genetic relatedness among lines. The proportion of variance explained by each principal component is shown, and scatter plots depict the relationships among the first three principal components (PC1, PC2, and PC3).

**Supplementary Fig. 7.** Quantile–quantile (Q–Q) plots of GWAS results for essential oil compound levels in *A. lancea*. Quantile–quantile (Q–Q) plots comparing observed and expected −log₁₀(p) values for genome-wide association analyses of atractylodin, hinesol, β-eudesmol, and atractylon.

**Supplementary Fig. 8.** Atractylon values stratified by genotype at the significant SNP. Boxplots showing (A) original atractylon content (mg g⁻¹) and (B) inverse-normal transformed atractylon values for the three genotypic classes (0, 1, and 2) of the SNP significantly associated with atractylon in the GWAS. Crosses indicate mean values. Sample sizes for each genotype are shown above the plots. No significant differences among genotypes were detected at the 5% significance level based on the Kruskal–Wallis test for original values and one-way ANOVA for inverse-normal transformed values.

**Supplementary Fig. 9.** Genome-wide distribution of SNP effect estimates obtained using the GBLUP (RR) model. Genome-wide distributions of SNP effect estimates for atractylodin, hinesol, β-eudesmol, and atractylon were estimated using the GBLUP (RR) model. SNP effects are plotted according to their physical positions along each chromosome. In this model, marker effects are assumed to follow a common normal distribution with homogeneous variance across all SNPs.

**Supplementary Fig. 10**. Genome-wide distribution of SNP effect estimates obtained using the BayesC model. Genome-wide distributions of SNP effect estimates for atractylodin, hinesol, β-eudesmol, and atractylon were estimated using the BayesC genomic prediction model. SNP effects are plotted according to their physical positions along each chromosome. BayesC allows a subset of SNPs to have zero effects, while estimating non-zero effects for the remaining markers.
